# HPLC-HRMS Global Metabolomics Approach for the Diagnosis of “Olive Quick Decline Syndrome” Markers in Olive Trees Leaves

**DOI:** 10.3390/metabo11010040

**Published:** 2021-01-08

**Authors:** Alberto Asteggiano, Pietro Franceschi, Michael Zorzi, Riccardo Aigotti, Federica Dal Bello, Francesca Baldassarre, Francesco Lops, Antonia Carlucci, Claudio Medana, Giuseppe Ciccarella

**Affiliations:** 1 Department of Molecular Biotechnology and Health Sciences, University of Torino, Via Pietro Giuria 5, 10125 Torino, Italy; alberto.asteggiano@unito.it (A.A.); michael.zorzi@unito.it (M.Z.); riccardo.aigotti@unito.it (R.A.); federica.dalbello@unito.it (F.D.B.); 2 Unit of Computational Biology, IASMA Research and Innovation Centre, Fondazione Edmund Mach via E. Mach, 1, 38010 San Michele all’ Adige, Italy; pietro.franceschi@fmach.it; 3 Biological and Environmental Sciences Department, UdR INSTM of Lecce University of Salento, Via Monteroni, 73100 Lecce, Italy; francesca.baldassarre@unisalento.it; 4 Institute of Nanotechnology, CNR NANOTEC, Consiglio Nazionale delle Ricerche, Via Monteroni, 73100 Lecce, Italy; 5 Department of Science of Agriculture, Food and Environment, University of Foggia, Via Napoli, 25, 71122 Foggia, Italy; francesco.lops@unifg.it (F.L.); antonia.carlucci@unifg.it (A.C.)

**Keywords:** olive quick decline syndrome, liquid chromatography, high-resolution mass spectrometry, metabolomics

## Abstract

Olive quick decline syndrome (OQDS) is a multifactorial disease affecting olive plants. The onset of this economically devastating disease has been associated with a Gram-negative plant pathogen called *Xylella fastidiosa* (Xf). Liquid chromatography separation coupled to high-resolution mass spectrometry detection is one the most widely applied technologies in metabolomics, as it provides a blend of rapid, sensitive, and selective qualitative and quantitative analyses with the ability to identify metabolites. The purpose of this work is the development of a global metabolomics mass spectrometry assay able to identify OQDS molecular markers that could discriminate between healthy (HP) and infected (OP) olive tree leaves. Results obtained via multivariate analysis through an HPLC-ESI HRMS platform (LTQ-Orbitrap from Thermo Scientific) show a clear separation between HP and OP samples. Among the differentially expressed metabolites, 18 different organic compounds highly expressed in the OP group were annotated; results obtained by this metabolomic approach could be used as a fast and reliable method for the biochemical characterization of OQDS and to develop targeted MS approaches for OQDS detection by foliage analysis.

## 1. Introduction

The year 2013 will be recorded as the annus horribilis for olive trees of the Salento Peninsula in south-eastern Italy [1]. Compared to other Italian regions, olive cultivation in Puglia (also known as Apulia) has a more significant spread, with the associated strong economic impact. For nearly a decade in the province of Lecce and the southern zones of the Brindisi and Taranto provinces [2], olive trees have been affected by a progressive disease that begins with foliage desiccation, quickly ending up with the death of the whole tree. This condition of unknown etiology was named “olive quick decline syndrome” (OQDS, known in Italy also as CoDiRO, Complesso del Disseccamento Rapido dell’Olivo) [3,4]. OQDS is a disease characterized by leaves yellowing and desiccation of twigs and small branches, which eventually results in tree death. In the early stages, symptoms appear on the apical parts of trees, and subsequently, they then extend to the rest of the crown, which acquires a burned-like aspect in the last stages of disease. To contrast OQDS and promote new growth, heavy pruning has always been applied as an agronomical control approach, but this strategy has proven ineffective [5]. The onset of this economically devastating disease has been associated with *Xylella fastidiosa* (Xf) infection. Xf is a formidable Gram-negative pathogen that has already caused enormous damage in the United States and South America [6]. These bacteria form biofilms inside xylem vessels, leading to their occlusion, which eventually impairs water and mineral salt uptake [7] in the apical regions of the plant organism. To avoid an uncontrolled spread of this pathogen, the European Union has introduced mandatory measures of containment mostly via the eradication of plants carrying the infected phenotypes and all the plants growing in their surroundings creating a buffer zone to protect the rest of the Italian Peninsula [8].

This bacterium has been proven to be responsible for the development of some other economically significant diseases, including Pierce’s disease of grapevine [9], leaf scorch of almond, oleander, and coffee [10], citrus variegated chlorosis, and other diseases of crop, forest, and landscape plants [11]. In the case of olive, damage may be aggravated by the presence of fungi of different genera, *Phaeoacremonium* and *Phaemoniella* in particular, but also *Pleumostomophora* and *Neofusicoccum*, which colonize and necrotize sapwood [2]. In order to develop less drastic strategies for containing the disease spread, researchers have studied bacterial homeostasis and its interaction with plants, investigating the role of different small molecules, such as minerals and phenolics [12,13,14]. Recently, Baldassarre et al. proposed a nanotechnological tool to contrast infection exploiting CaCO_3_ nanocarriers [15]. To date, however, except for quarantine and eradication, there are no ways to fight this infection.

An early disease detection method also able to characterize the biochemistry of the infection and of the plant response would certainly be an efficient weapon to contrast Xylella’s spread. Unfortunately, to date, it is possible to detect the presence of Xf in olive trees only by serological (ELISA) and molecular (PCR) methods [16,17]. Both methods are sensitive, but they cannot be directly used to characterize the complex phenomena occurring during infection.

Metabolomics is one of the most powerful tools for characterizing molecular profiles and their correlation with physiological or pathological conditions in biological or natural samples [18]. The main goal of this work is to propose an untargeted metabolomics approach combined with an appropriate analytical method to identify OQDS molecular markers able to discriminate between healthy and infected trees. This global metabolomics approach is potentially able to measure the levels of thousands of metabolites in a single analysis and suits perfectly for our purposes thanks to the comprehensive information on the composition of a metabolite pool it can provide [19]. Beside the possibility of characterizing the biological phenomena occurring during the infection, the outcomes of this type of investigation could also be used to identify a panel of biomarkers that could then be exploited by an analytical laboratory through targeted metabolomics approaches. These methods require a priori knowledge of metabolites of interest but are cheaper, faster, and more accurate if compared to untargeted ones [20].

## 2. Results

**Figure 1:**
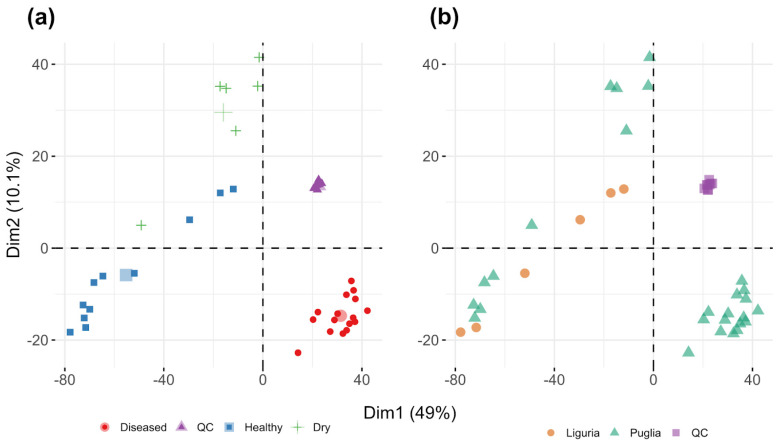
PCA score plots for all the samples analyzed: (**a**) bidimensional separation highlighting the infection state: red circles represent the diseased population, violet triangles the quality control (QC) samples, light blue squares the healthy samples and green crosses the healthy dried samples. For each sample class, the bigger symbol (circle, triangle, square and cross) represents its center of gravity. (**b**) bidimensional separation highlighting the origin factor; orange circles represent samples originating from Liguria, green triangles are used for samples originating from Puglia while violet squares for QC pooled samples.

Principal component analysis was performed to understand the factors which account for the larger fraction of variance in the dataset and the results are shown in Figure 1. 

The score plot shows the distribution of the sample points in the PC1 vs. PC2 planes which account for a significant fraction of the overall variance of the dataset (50.1%).

**Figure 2:**
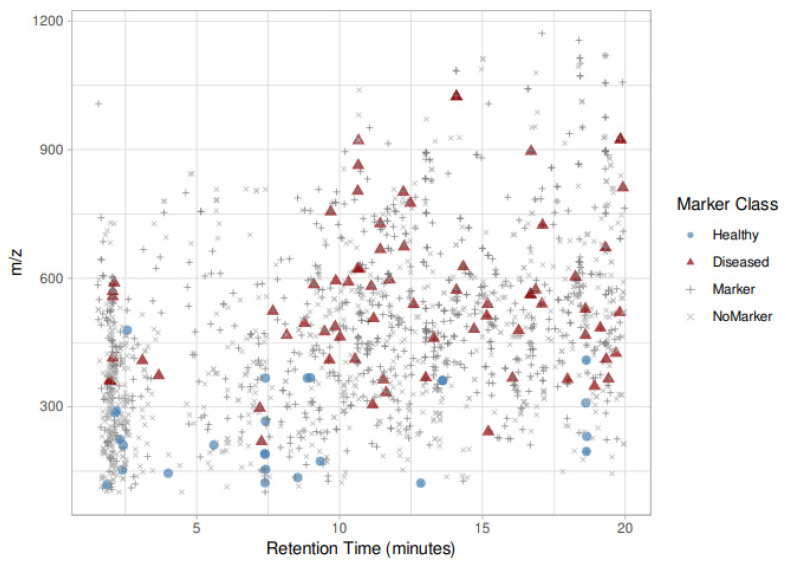
Two-dimensional map of features discovered, visually separated for their retention time (*x*-axis) and their *m*/*z* ratio (*y*-axis); blue and red colored dots represent, respectively, healthy and diseased state markers (77 and 26).

The results of the univariate statistical analysis on the full set of features extracted from the raw data substantially confirmed the marked difference between healthy and diseased samples. Out of the overall set of 3086 features, 1770 showed a significantly different concentration in healthy and diseased samples (*p* < 0.01 after Bonferroni correction). The two-dimensional map of the identified features is presented in Figure 2. In order to prioritize the annotation phase, we decided to focus on the features showing the higher contrast in the two sample classes, deciding to classify potential healthy–infected discrimination markers all the significant features which show a change of their median intensity from the first to the fourth quartile in the healthy-infected sample classes. 

With this criterion, out of the 1770 significant features, 118 were classified as infection state markers and 37 as healthy state markers. Their position in the *mz*/rt plane is also highlighted in Figure 2; Tables S1 and S2 report, respectively, infected and healthy discriminating features.

HPLC-HRMS and tandem MS analysis allowed for the identification of key molecules that are expressed in a significantly different manner between the groups of healthy and sick leaves. These features could then be used as putative markers to discriminate the HP and OP samples.

**Figure 3:**
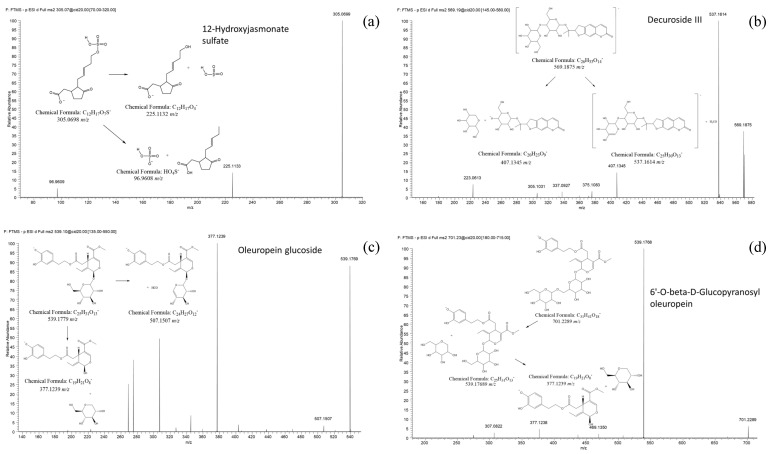
MS^2^ spectra of some annotated features together with their fragmentation patterns: 12-Hydroxyjasmonate sulfate (**a**), Decuroside III (**b**), Oleuropein glucoside (**c**) and 6’-O-beta-D-Glucopyranosyl-oleuropein (**d**).

**Figure 4:**
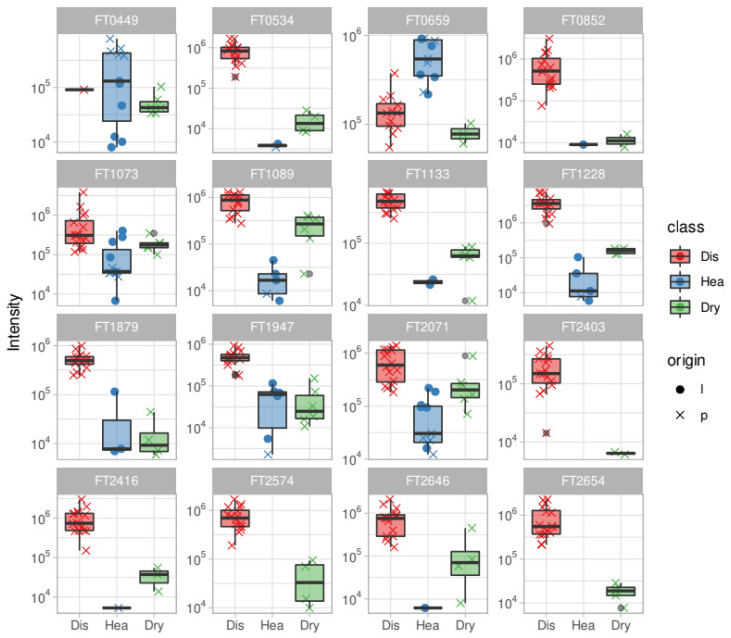
Boxplots for the annotated features; abundances are reported for each class together with the origin factor. Red boxes represent the diseased samples, blue boxes the healthy samples and green boxes the dried samples.

**Figure 5:**
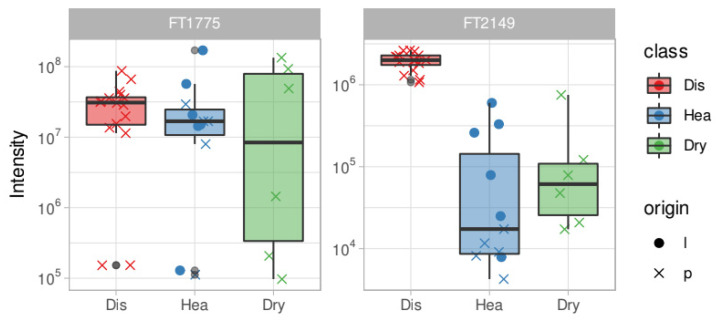
Boxplots for reported O.e species-specific markers Red boxes represent the diseased samples, blue boxes the healthy samples and green boxes the dried samples.

Among all the discriminating features we annotated, thanks to the HPLC-HRMS and tandem HRMS approach, 16 molecules and two additional molecules reported to be expressed in *Olea europaea* leaves (O.e Marker) [21] were listed as well. The details of chemical identification are reported in Table 1, Figure 3 shows some examples of tandem MS experiments reporting the fragmentation pathways of some annotated molecules. While their intensity profile is presented as a boxplot in Figure 4 and Figure 5. 

Boxplots show the relative abundance of features in the different classes of samples (infected, healthy, and healthy desiccated), allowing for interpreting and inferring their distribution related to the sample state of health. 

In Figure 5, the boxplot reports the features considered as O.e markers [21].

## 3. Discussion

To date, there are no literature reports of the use of a HPLC-HRMS untargeted method to discover the early infection markers for *Xylella fastidiosa*-related OQDS. However, several untargeted metabolomics approaches were developed for the characterization and valorization of different *Olea europaea* cultivars and origins [22,23] and their oils [24,25].

### 3.1. HPLC-HRMS Analytical Method Development

The analytical chromatographic method proposed was developed with the aim of obtaining the best separation of analytes in the shortest time possible to maximally lower the inter-run retention time deviation of the molecules, thus also minimizing the peak-picking errors. The HPLC method chosen consisted in a multi-step gradient which was empirically time-optimized during the experimental design to better separate both high hydrophilic compounds such as organic acids and glycosylated metabolites up to lipophilic phytosterols. MS acquisition was performed in the negative ion mode as it is less affected by background noise than the positive ion mode [26]. An example of TIC chromatograms is reported in Figure S1.

### 3.2. Sample Two-Dimensional Clustering

In Figure 1a, three different sample clusters are clearly visible. The tighter cluster is composed of QC samples (violet) and its low spread confirms the low variability of the analytical pipeline. As expected, infected samples (red dots) show a higher variability, but they are otherwise a well-separated group. The larger and most diverse cluster is composed of healthy and desiccated samples, which are only partially separated in this bivariate projection. Interestingly, desiccated samples are positioned far away from the diseased group, indicating that desiccation alone is not responsible for the metabolic separation between healthy and diseased samples. It is worth noting that healthy and diseased samples are separated along the direction of larger variance (PC1, 49%) and this indicates that their difference is the most important factor affecting the overall variability of the dataset.

As discussed in the introduction, regionality might also play a role in sample clustering, acting as a potential confounder. For this purpose, the association of the samples to their specific origin is shown in Figure 1b. Healthy and desiccated samples from Puglia and Liguria are clearly distributed homogeneously inside the “healthy” cluster. These data suggest that also the origin of samples (Liguria vs. Puglia) does not play a prominent role in driving the observed differentiation between healthy and diseased samples. QC samples, obtained by pooling equal aliquots of every single sample, show a very condensed grouping in the PCA score plot. Their variance on both PCA axes is so narrow that we can assess that there is no evident analytical drifting that could affect our analytical method.

### 3.3. Features Annotation

Most of the molecules annotated belong to the class of plants secondary metabolites such as 12-hydroxy jasmonate sulfate, a molecule discovered in *Arabidopsis thaliana* which derives from jasmonic acid, which has a signaling role mediating diverse developmental processes and plant defense responses. The potential finding of this molecule suggests that the infected plant builds a defense response against the stress condition [27].

Nodakenin is a coumarin compound firstly found in the root of *Angelica gigas* and, as a coumarin, its function is to discourage herbivores and insect species from eating the plant [28]. Together with nodakenin, another coumarin, decuroside, has been MS/MS-annotated. Physalin, first discovered in *Physalis alkekengi,* is a phytosteroidal molecule [29]; this molecule has accentuated antimicrobial and antibacterial effects [30] and its presence can be attributed to the plant’s stress condition. Another annotated compound: marchantin A, a macrocyclic bis-benzyl ether isolated from *Marchantia emarginata*, is known for its antiprotozoal activity, suggesting that the plant may also defend from concurrent protozoal infections [31]. 6’-O-beta-D-glucopyranosyloleuropein is a molecule expressed in the Oleaceae plant family and belongs to the class of secoiridoid diglucosides [32], similar to the widely known secoiridoid glucoside oleuropein which has also been annotated as a proof of consistency for this work since it is a highly species-specific marker for *Olea europaea*, together with rutin.

Several other features were annotated by using only the HRMS discrimination power since their intensity was not able to trigger the dependent data acquisition, such as niazicinin A, a phenolic glucoside; vismione D, a molecule with antiprotozoal activity; the flavone ovaliflavanone; patuletin 3-(4’’-acetylrhamnoside)-7-(2’’’-acetylrhamnoside), a flavone from the aerial parts of *Echinacea angustifolia* [33]; aldosecologanin, a iridoid glycoside isolated from *Lonicera japonica* [34]; rhamnazin 3-rhamninoside, a flavonoid glycoside with anti-fungal and antibacterial activity [35]; and purpureaside C, a phenolic glycoside with an antimicrobial effect [36]. 

On the other hand, we annotated some features mostly abundant in healthy samples such as the acridone alkaloid grandisine III isolated in *Citrus grandis* [37] and isomurrayazoline, a carbazole alkaloid found in *Murraya koenigii* [38].

### 3.4. Feature Abundance Class-Related Variability

Figure 4 shows abundance boxplots of the annotated features in each class. Most of the features identified appear to be up-regulated in infected samples and it is interesting to notice the intensity change in each class; the desiccation seems to play a role in incrementing the signal of a given feature, probably by increasing its concentration, reducing the amount of water. This phenomenon is, however, not as intense as what happens in the infected class.

Regionality is a known factor in differentiating the metabolome of *Olea europaea* trees and leaves [21]; in this case, it seems to play a role with some features such as FT2071 and FT1133, where in these, we can observe a different intensity distribution in healthy samples from Liguria and Puglia. These features, in addition to being reliable infection markers, can be used also as regionality markers, giving strength to this global metabolomic approach itself. Oleuropein (FT1775) shows a similar abundance distribution in healthy and desiccated classes except for some outliers which are, however, comparable with the two classes; in the case of FT2149: rutin, there is an appreciable origin-related variability in healthy samples (Figure 5).

## 4. Materials and Methods 

### 4.1. Chemicals and Materials

Ammonium acetate, formic acid LC-MS grade, catechin, and galangine were purchased from Sigma Aldrich (Milan, Italy). Acetonitrile and methanol solvents were purchased from VWR Italia (Milan, Italy) and were used without any further purification treatment. All the aqueous solutions were prepared by using ultrapure water (Merck Millipore MilliQ ™, Darmstadt, Germany).

### 4.2. Sampling Protocol of Plant Samples

Plant extracts considered in our investigation belonged to four distinguished groups: “Puglia infected”, “Liguria healthy”, “Puglia healthy”, and “Puglia desiccated”. It was impossible to collect Liguria infected samples since, to date, there is no evidence of Xf infection in any Italian region other than Puglia. Puglia infected samples were harvested from plants infected by *Xylella fastidiosa*; their infection state was confirmed by an antibody ELISA kit test. In total, 17 healthy samples (HP) comprehending 6 desiccated samples (DHP) and 15 samples with OQDS symptoms (OP) were collected directly from olive trees. The two groups of healthy samples were extracted from healthy olive trees grown in Pornassio (Liguria region, Italy) and Salento (Puglia region, Italy). Finally, desiccated samples were collected from a desiccated branch of a healthy olive tree; Table 2 reports the state, origin, and the number of samples.

This specific experimental design was decided to compare the effect of the infection with potential confounding factors such as the farming locations (Puglia or Liguria regions, Italy) or the sample dryness, since infected samples are in desiccated form. 

### 4.3. Quality Controls Setting

Quality controls (QCs) are created to assess and ensure that the analytical method created is performed appropriately and meets the criteria defined a priori. In our case, the QC sample was a pooled sample in which a small aliquot (20 µL) of each extracted sample under analysis was mixed in a 10-mL tube. By this way, the pooled QC created represents the matrix as the metabolites’ composition of Xylella-infected samples. 

Frequency of QC injections [39] was set according to earlier publications, focusing particularly upon [40]. HP and OD samples were injected in randomized run order in the same batch. QC injections were performed in each of the 5 samples. Additionally, at the beginning of the analysis, 10 consecutive injections of QC samples were performed to prime the column. 

### 4.4. Sample Harvesting and Preparation

An amount of 600 g of leaves was harvested from each tree; 10 of them were sampled and immediately shock-frozen with liquid nitrogen to block all metabolic processes and transferred to the laboratory for the extraction. 

It is fundamental to quench the metabolism as soon as possible, and shock freezing using liquid nitrogen is the most common and efficient method to inactivate the metabolism and preserve all the metabolites. 

### 4.5. Extraction Protocol of Plant Samples and QCs Sample Generation

Leaves were flash frozen in liquid nitrogen and then manually grounded with a pestle and a mortar (pre-cooled and filled with liquid nitrogen). An amount of 300 mg of fine powder was extracted with 1.2 mL of 70% aqueous methanol in 1.5 mL Eppendorf tubes, sonicated for 15 min, and centrifuged at 13,680× *g* for 20 min. An amount of 500 μL of the supernatants was transferred in new Eppendorf tubes and the solvent evaporated under a stream of nitrogen. The addition of 1 mL of water/acetonitrile 50:50 (*v*/*v*) to the dry extract was followed by sonication (15 min) and centrifugation (13,680× *g*, 20 min). 

Before conducting the analyses, each sample was spiked with two different internal standards: catechin (289.0790 *m*/*z* negative ion mode), and galangine (269.0528 *m*/*z* negative ion mode). The use of two different internal standards (eluting by the analytical column at different retention times) is a fundamental condition for having good results during the data alignment process. All injections were performed in the same batch.

### 4.6. HPLC-ESI-LTQ Orbitrap Parameters

Analyses were performed on a HPLC-ESI HRMS. Instrument setup for all the analyses consisted of a Dionex Ultimate 3000 HPLC system equipped with a solvent vacuum degasser (Thermo Scientific, Milan, Italy) coupled with a high-resolution mass spectrometer, LTQ-Orbitrap (Thermo Scientific, Milan, Italy), through an electrospray ionization (ESI) interface.

Chromatograms were recorded using Thermo Xcalibur 3.0 software (Rev. SP1 1160). A Gemini NX-C18 column from Phenomenex (Gemini NX-C18, 2.0 × 150 mm, 3.0 µm, 110 Å, Phenomenex, Bologna, Italy) was used to obtain chromatographic separation of the extracts. Mobile phase was ammonium acetate 0.005 M in ultrapure water (C) and acetonitrile (B). Gradient elution was set as follows: linear gradient from 5 to 20% B in 8 min, then 20% B held for 4 min, linear gradient from 20 to 30% B from 12 to 20 min, then in 2 min reaches 100% B; in 1 min the percentage of acetonitrile returns to initial conditions: (5%) and it is held for 10 min (from 23 to 33 min) to ensure the correct equilibration of the column at the initial condition percentages. The flow rate was set at 200 μL/min. Global run time was 33 min. The column temperature was set at 25 °C. Sample injection volume was 10 μL. 

The HRMS system operated in the negative ionization mode. ESI tuning parameters were set as follows: capillary voltage was −13 V (ESI-); tube lens was set at −36 V (ESI-); source voltage was set to 3.5 kV (ESI-); sheath gas and aux gas flow rate were, respectively, 35 and 20 arbitrary units in both methods; spray current was set at 0.05 µA; capillary temperature was 270 °C during all the analyses. The mass spectrometer operated in full-scan mode in the range 100–1200 *m*/*z* (ESI-), with a resolution of 30,000 in FTMS mode. Tandem mass (MS/MS) experiments were automatically performed in the range 100–1200 *m*/*z* (ESI-), using the automatic dependent scan function. Collision energy was set at 30 (arbitrary units) for all the MS acquisitions. All spectra were acquired in centroid mode. Xcalibur 3.0 software (Rev. SP1 1160, Thermo Scientific, Bremen, Germany) was used both for acquisition and for elaboration and calculation.

### 4.7. Data Processing and Statistical Analysis

Raw LC-MS data files were converted into mzXML using the open source software ProteoWizard—MSConvert [41].

Data pre-processing was performed with XCMS [42].

Parameters applied for the processing were set as follows: centWave for feature detection (peakwidth = c (20, 80), prefilter = c (3, 50000), ppm = 10); retention time correction was performed with obiwarp (binSize = 0.6); peak matching across the samples (minFraction = 0.8, bw = 40). Before statistical analysis, missing peaks were imputed by applying the FillMissingPeaks algorithm available in XCMS. Statistical analyses were performed in R on the matrix constructed by extracting the maximum value of the intensity measured on each feature chromatographic peak (maxo). Log transformation was used to correct for the expected heteroskedasticity of metabolomics data. 

Due to the strong differences in the samples belonging to the different classes, no sample normalization was performed. To pinpoint the features showing the stronger contrast, a two-stage strategy was applied:

1. Kruskall–Wallis test was applied to identify the features showing a significant difference between HP and OP samples (*p* < 0.01 after Bonferroni correction).
2. Significant features were then ranked on the bases of their median intensity in the two sample classes.
3. The potential list of infection biomarkers was selected:
  a- By considering the features present in the top quartile of the ranked list for OP and in the lower quartile of the HP list.
  b- By considering the features present in the top quartile of the ranked list for HP and in the lower quartile of the OP list.

Tables S1 and S2 report the discriminating features found together with their averaged *m*/*z* ratio and retention times.

### 4.8. Metabolites Identification

Identification of single metabolites was achieved by MS/MS spectrum matching with available online databases such as MoNA and UNPD [43], and the MetFrag online tool [44]. When a tandem MS experiment was not present, we attempted molecular recognition by the accurate *m*/*z* signal which was converted to a putative molecular formula by means of Xcalibur Qual browser 3.0 software (Rev. SP1 1160, Thermo Scientific, Bremen, Germany) or the Metlin [45,46] online database. Proposed formulas were ranked for their Δ-ppm (0 to 6 ppm max); other applied constraints were the included atom elements: C: 0 to 50, O: 0 to 30, N: 0 to 10, H: 0 to 100, S: 0 to 5, P: 0 to 5; and RDB equivalent: −1 to 30 (for Xcalibur Qual browser only). As already mentioned, all analytes detected by our untargeted approach are classified as per MSI guidelines (Metabolomic Standard Initiative) [47].

## 5. Conclusions

An untargeted metabolomics approach was applied to olive leaves samples with the aim to understand the main differences between healthy plants and plants with OQDS-like symptoms. To this end, we followed an extraction procedure with aqueous methanol and developed a simple, accurate, high-resolution mass-based analysis method that could detect the broadest range of metabolites. 

Results of multivariate analysis show a clustering of two pools of samples (HP vs. OP) based on two principal components (PC1 and PC2). Notably, the addition of Ligurian samples with different regionality factors and of desiccated samples proved the robustness of the method which is still capable of clustering healthy and infected samples on the first PCA axis. In addition, eighteen different organic compounds, among which 14 were highly expressed in the OP group, were annotated. 

These results should pave the way for a targeted and feasible analytical approach aimed at the detection of early infection state-related molecules for all the research and routine laboratories who cannot afford a global metabolomics instrumentation setup. However, a truly comprehensive analysis of the plant metabolite pool is not easily feasible due to the large number of primary and secondary metabolites in any given plant species. Each analytical technology has advantages and limitations, and not one can cover the whole metabolome due to the chemical diversity of metabolites and their broad dynamic range in cellular abundance [48]. Consequently, different extraction techniques and combinations of analytical methods should be employed in attempts to achieve adequate metabolite coverage [49].

In future, the primary goal is to obtain a more significant number of features by implementing diverse extraction methods and by merging results upcoming from MS to other techniques more oriented to identification and characterization such as NMR spectroscopy.

More in-depth and more accurate knowledge of the olive metabolome and its infection-related differences can be provided by reiterating the analysis of different years to verify whether the data obtained by analyzing samples belonging to a single vintage remain unchanged or not by analyzing olive trees of different years.

## Figures and Tables

**Table 1:** Annotated molecules; each feature is specified whenever it is a health or infection marker or a reported species-specific marker (O.e Marker) for *Olea europaea*. The table also reports the double bond equivalent (DBE) and the mass accuracy shift in ppm.

Feature ID	Healthy–Infected	*m*/*z* [M − H]^−^	R.t. (min)	MSI Level	Fragments (Intensity)	Proposed Formula	Proposed Name ^(^*^)^	DBE	Δ (PPM)
FT0449	Healthy	286.0732	2.1	3		C_15_H_13_NO_5_	Grandisine III ^(b)^ CAS: 53421-39-9	10	5.79
FT0534	Infected	305.0698	11.2	2	305.0698 (100) 225.1133(14) 96.9608(5)	C_12_H_18_O_7_S	12-Hydroxy Jasmonate sulfate ^(a)^ CID: 44815853	4.5	0.92
FT0659	Healthy	330.1871	25.1	3		C_23_H_25_NO	Isomurrayazoline ^(b)^ CAS: 85547-20-2	12	3.94
FT0852	Infected	368.1366	13.0	3		C_17_H_23_NO_8_	Niazicinin ^(b)^ CID: 101920262	7	5.64
FT1073	Infected	407.1346	3.1	2	337.0925(100) 305.1029 (90) 375.1081 (48) 407.1344 (41)151.0405 (20)	C_20_H_24_O_9_	Nodakenin ^(a)^ CID: 73191	9.5	0.95
FT1089	Infected	409.2005	9.6	3		C_25_H_30_O_5_	Vismione D ^(b)^ CID: 5281573	11	2.37
FT1133	Infected	419.1847	13.4	3		C_26_H_28_O_5_	Ovaliflavanone D ^(b)^ CID: 42607825	13	2.52
FT1228	Infected	439.1536	21.1	2	393.1762(100) 197.0821 (35)	C_28_H_24_O_5_	Marchantin A ^(a)^ CID: 88418-46-6	17.5	0.39
FT1775	O.e Marker	539.1769	20.9	2	377.1239 (100) 507.1507 (5)	C_25_H_32_O_13_	Oleuropein glucoside ^(c)^ CID: 5281544	10	0.79
FT1879	Infected	557.2002	2.1	2	513.2335 (100) 345.1185 (21) 227.1288 (8)	C_29_H_34_O_11_	Physalin ^(a)^ Metlin ID: 89909	13.5	0.46
FT1947	Infected	569.1844	20.1	2	537.1612 (100) 403.1242 (95) 569.1873 (46) 407.1344 (13)	C_26_H_34_O_14_	Decuroside III ^(a)^ CAS: 96638-81-2	10.5	1.69
FT2071	Infected	593.1404	18.2	2	593.1507 (100) 285.0403 (98)	C_27_H_30_O_15_	Isoorientin rhamnoside ^(a)^ CID: 16126794	13	3.79
FT2149	O.e Marker	609.1458	14.5	3		C_27_H_30_O_16_	Rutin ^(c)^ CID: 5280805	13	0.38
FT2403	Infected	701.2271	11.7	2	539.1768 (100) 377.1238 (5)	C_31_H_42_O_18_	6’-O-beta-D-Glucopyranosyl-oleuropein ^(a)^ CID: 102078602	11	3.13
FT2416	Infected	707.1863	24.7	3		C_32_H_36_O_18_	Patuletin 3-(4’’-acetylrhamnosid)7-(2’’’-acetylrhamnoside) ^(b)^ CID: 44259840	15	5.73
FT2574	Infected	757.2582	21.9	3		C_34_H_46_O_19_	Aldosecologanin ^(b)^ CID: 10908841	12	3.58
FT2646	Infected	783.2375	24.8	3		C_35_H_44_O_20_	Rhamnazin 3-rhamninoside ^(b)^ CID: 44259609	14	3.51
FT2654	Infected	785.2533	24.8	3		C_35_H_46_O_20_	Purpureaside ^(b)^ CID: 11953944	13	3.76

^(^*^)^ Putative annotation obtained by: ^(a)^ Metfrag, MoNA, ^(b)^ Metlin, ^(c)^ Xcalibur Qual Browser.

**Table 2:** Distribution of samples, state, and origin.

Healthy		Infected
Normal (HP)	Desiccated (DHP)	Desiccated (OP)
11	6	16
5 Puglia, 6 Liguria	6 Puglia	16 Puglia

## Data Availability

Data is contained within the article or supplementary material.

## Supplementary Materials

The following are available online at https://www.mdpi.com/2218-1989/11/1/40/s1, Figure S1: Infected (OP), desiccated (DHP), and healthy (HP) samples’ total ion current (TIC) chromatograms in the full acquisition time range, Table S1: All infected discriminating features reported together with Rt (min), Table S2: All healthy-discriminating features reported together with Rt (min).

## References

[1] D’Onghia A.M., Digiaro M., Brunel S., Valentini F. (2017). Xylella Fastidiosa & the Olive Quick Decline Syndrome (OQDS) A Serious Worldwide Challenge for the Safeguard of Olive Trees.

[2] Martelli G.P., Boscia D., Porcelli F., Saponari M. (2016). The olive quick decline syndrome in south-east Italy: A threatening phytosanitary emergency. Eur. J. Plant Pathol..

[3] Nigro F., Boscia D., Antelmi I., Ippolito A. (2013). Fungal species associated with a severe decline of olive in southern Italy. J. Plant Pathol..

[4] Saponari M., Loconsole G., Cornara D., Yokomi R.K., De Stradis A., Boscia D., Bosco D., Martelli G.P., Krugner R., Porcelli F. (2014). Infectivity and Transmission of Xylella fastidiosa by *Philaenus spumarius* (Hemiptera: *Aphrophoridae*) in Apulia, Italy. J. Econ. Entomol..

[5] Cariddi C., Saponari M., Boscia D., De Stradis A., Loconsole G., Nigro F., Porcelli F., Potere O., Martelli G.P. (2014). Isolation of Xylella fastidiosa strain infecting olive and oleander in Apulia, Italy. J. Plant Pathol..

[6] Colnaghi Simionato A.V., da Silva D.S., Lambais M.R., Carrilho E. (2007). Characterization of a putativeXylella fastidiosa diffusible signal factor by HRGC-EI-MS. J. Mass Spectrom..

[7] Marques L.L.R., Ceri H., Manfio G.P., Reid D.M., Olson M.E. (2002). Characterization of Biofilm Formation by Xylella fastidiosa In Vitro. Plant Dis..

[8] Abbott A. (2018). Italy’s olive crisis intensifies as deadly tree disease spreads. Nature.

[9] Hopkins D.L., Purcell A.H. (2002). Xylella fastidiosa: Cause of Pierce’s Disease of Grapevine and Other Emergent Diseases. Plant Dis..

[10] Hernandez-Martinez R., Costa H.S., Dumenyo C.K., Cooksey D.A. (2006). Differentiation of Strains of Xylella fastidiosa Infecting Grape, Almonds, and Oleander Using a Multiprimer PCR Assay. Plant Dis..

[11] Janse J.D., Obradovic A. (2010). Xylella fastidiosa: Its biology, diagnosis, control and risks. J. Plant Pathol..

[12] Maddox C.E., Laur L.M., Tian L. (2010). Antibacterial Activity of Phenolic Compounds Against the Phytopathogen Xylella fastidiosa. Curr. Microbiol..

[13] Navarrete F., De La Fuente L. (2014). Response of Xylella fastidiosa to Zinc: Decreased Culturability, Increased Exopolysaccharide Production, and Formation of Resilient Biofilms under Flow Conditions. Appl. Environ. Microbiol..

[14] Wang P., Lee Y., Igo M.M., Roper M.C. (2017). Tolerance to oxidative stress is required for maximal xylem colonization by the xylem-limited bacterial phytopathogen, Xylella fastidiosa. Mol. Plant Pathol..

[15] Baldassarre F., De Stradis A., Altamura G., Vergaro V., Citti C., Cannazza G., Capodilupo A.L., Dini L., Ciccarella G. (2020). Application of calcium carbonate nanocarriers for controlled release of phytodrugs against Xylella fastidiosa pathogen. Pure Appl. Chem..

[16] Bextine B., Tuan S.-J., Shaikh H., Blua M., Miller T.A. (2004). Evaluation of Methods for Extracting Xylella fastidiosa DNA from the Glassy-Winged Sharpshooter. J. Econ. Entomol..

[17] Loconsole G., Potere O., Boscia D., Altamura G., Djelouah K., Elbeaino T., Frasheri D., Lorusso D., Palmisano F., Pollastro P. (2014). Detection of Xylella fastidiosa in olive trees by molecular and serological methods. J. Plant Pathol..

[18] Zhou B., Xiao J.F., Tuli L., Ressom H.W. (2012). LC-MS-based metabolomics. Mol. BioSyst..

[19] Patti G.J., Yanes O., Siuzdak G. (2012). Metabolomics: The apogee of the omics trilogy. Nat. Rev. Mol. Cell Biol..

[20] Zhang X., Zhu X., Wang C., Zhang H., Cai Z. (2016). Non-targeted and targeted metabolomics approaches to diagnosing lung cancer and predicting patient prognosis. Oncotarget.

[21] Di Donna L., Mazzotti F., Naccarato A., Salerno R., Tagarelli A., Taverna D., Sindona G. (2010). Secondary metabolites of Olea europaea leaves as markers for the discrimination of cultivars and cultivation zones by multivariate analysis. Food Chem..

[22] Kritikou E., Kalogiouri N.P., Kolyvira L., Thomaidis N.S. (2020). Target and Suspect HRMS Metabolomics for the Determination of Functional Ingredients in 13 Varieties of Olive Leaves and Drupes from Greece. Molecules.

[23] Guodong R., Xiaoxia L., Weiwei Z., Wenjun W., Jianguo Z. (2017). Metabolomics reveals variation and correlation among different tissues of olive (*Olea europaea* L.). Biol. Open.

[24] Stilo F., Liberto E., Reichenbach S.E., Tao Q., Bicchi C., Cordero C. (2019). Untargeted and Targeted Fingerprinting of Extra Virgin Olive Oil Volatiles by Comprehensive Two-Dimensional Gas Chromatography with Mass Spectrometry: Challenges in Long-Term Studies. J. Agric. Food Chem..

[25] Sales C., Portolés T., Johnsen L.G., Danielsen M., Beltran J. (2019). Olive oil quality classification and measurement of its organoleptic attributes by untargeted GC–MS and multivariate statistical-based approach. Food Chem..

[26] Liigand P., Kaupmees K., Haav K., Liigand J., Leito I., Girod M., Antoine R., Kruve A. (2017). Think Negative: Finding the Best Electrospray Ionization/MS Mode for Your Analyte. Anal. Chem..

[27] Gidda S.K., Miersch O., Levitin A., Schmidt J., Wasternack C., Varin L. (2003). Biochemical and Molecular Characterization of a Hydroxyjasmonate Sulfotransferase from Arabidopsis thaliana. J. Biol. Chem..

[28] Link K.P. (1959). The Discovery of Dicumarol and Its Sequels. Circulation.

[29] Matsuura T., Kawai M., Nakashima R., Butsugan Y. (1970). Structures of physalin A and physalin B, 13,14-seco-16,24-cyclo-steroids from Physalis alkekengi var. Francheti. J. Chem. Soc. C Org..

[30] Januário A.H., Filho E.R., Pietro R.C.L.R., Kashima S., Sato D.N., França S.C. (2002). Antimycobacterial physalins from Physalis angulata L. (*Solanaceae*). Phytother. Res..

[31] Jensen S., Omarsdottir S., Bwalya A.G., Nielsen M.A., Tasdemir D., Olafsdottir E.S. (2012). Marchantin A, a macrocyclic bisbibenzyl ether, isolated from the liverwort Marchantia polymorpha, inhibits protozoal growth in vitro. Phytomedicine.

[32] Huang Y.-L., Oppong M.B., Guo Y., Wang L.-Z., Fang S.-M., Deng Y.-R., Gao X.-M. (2019). The Oleaceae family: A source of secoiridoids with multiple biological activities. Fitoterapia.

[33] Lin L., Qiu S., Lindenmaier M., He X., Featherstone T., Cordell G.A. (2002). Patuletin-3-O-Rutinoside from the Aerial Parts of Echinacea angustifolia. Pharm. Biol..

[34] Machida K., Sasaki H., Iijima T., Kikuchi M. (2002). Studies on the Constituents of Lonicera Species. XVII. New Iridoid Glycosides of the Stems and Leaves of Lonicera japonica THUNB. Chem. Pharm. Bull..

[35] Azizah M., Pripdeevech P., Thongkongkaew T., Mahidol C., Ruchirawat S., Kittakoop P. (2020). UHPLC-ESI-QTOF-MS/MS-Based Molecular Networking Guided Isolation and Dereplication of Antibacterial and Antifungal Constituents of Ventilago denticulata. Antibiotics.

[36] Molnár J., Gunics G., Mucsi I., Koltai M., Petri I., Shoyama Y., Matsumoto M., Nishioka I. (1989). Antimicrobial and immunomodulating effects of some phenolic glycosides. Acta Microbiol. Hung..

[37] Tian-Shung W., Chang-Sheng K., Furukawa H. (1983). Acridone alkaloids and a coumarin from Citrus grandis. Phytochemistry.

[38] Bhattacharya L., Roy S.K., Chakraborty D.P. (1982). Structure of the carbazole alkaloid isomurrayazoline from Murraya koenigii. Phytochemistry.

[39] Kamleh M.A., Ebbels T.M.D., Spagou K., Masson P., Want E.J. (2012). Optimizing the Use of Quality Control Samples for Signal Drift Correction in Large-Scale Urine Metabolic Profiling Studies. Anal. Chem..

[40] Saigusa D., Okamura Y., Motoike I.N., Katoh Y., Kurosawa Y., Saijyo R., Koshiba S., Yasuda J., Motohashi H., Sugawara J. (2016). Establishment of Protocols for Global Metabolomics by LC-MS for Biomarker Discovery. PLoS ONE.

[41] Chambers M.C., Maclean B., Burke R., Amodei D., Ruderman D.L., Neumann S., Gatto L., Fischer B., Pratt B., Egertson J. (2012). A cross-platform toolkit for mass spectrometry and proteomics. Nat. Biotechnol..

[42] Smith C.A., Want E.J., O’Maille G., Abagyan R., Siuzdak G. (2006). XCMS: Processing Mass Spectrometry Data for Metabolite Profiling Using Nonlinear Peak Alignment, Matching, and Identification. Anal. Chem..

[43] Gu J., Gui Y., Chen L., Yuan G., Lu H.-Z., Xu X. (2013). Use of Natural Products as Chemical Library for Drug Discovery and Network Pharmacology. PLoS ONE.

[44] Ruttkies C., Schymanski E.L., Wolf S., Hollender J., Neumann S. (2016). MetFrag relaunched: Incorporating strategies beyond in silico fragmentation. J. Cheminform..

[45] Tautenhahn R., Patti G.J., Rinehart D., Siuzdak G. (2012). XCMS Online: A Web-Based Platform to Process Untargeted Metabolomic Data. Anal. Chem..

[46] Tautenhahn R., Cho K., Uritboonthai W., Zhu Z., Patti G.J., Siuzdak G. (2012). An accelerated workflow for untargeted metabolomics using the METLIN database. Nat. Biotechnol..

[47] Fiehn O., Robertson D., Griffin J., vab der Werf M., Nikolau B., Morrison N., Sumner L.W., Goodacre R., Hardy N.W., Taylor C. (2007). The metabolomics standards initiative (MSI). Metabolomics.

[48] Wang S., Alseekh S., Fernie A.R., Luo J. (2019). The Structure and Function of Major Plant Metabolite Modifications. Mol. Plant.

[49] Jorge T.F., Rodrigues J.A., Caldana C., Schmidt R., van Dongen J.T., Thomas-Oates J., António C. (2016). Mass spectrometry-based plant metabolomics: Metabolite responses to abiotic stress. Mass Spectrom. Rev..

