# Supplementary material for: HPLC-HRMS Global Metabolomics Approach for the Diagnosis of “Olive Quick Decline Syndrome” Markers in Olive Trees Leaves
Source: Metabolites. 2021 Jan 8;11(1):40. doi: 10.3390/metabo11010040 (PMC7827768; doi:10.3390/metabo11010040)
Supplement: Supplementary file 1 [file metabolites-11-00040-s001.pdf]

Metabolites-1043104 - Supplementary Materials

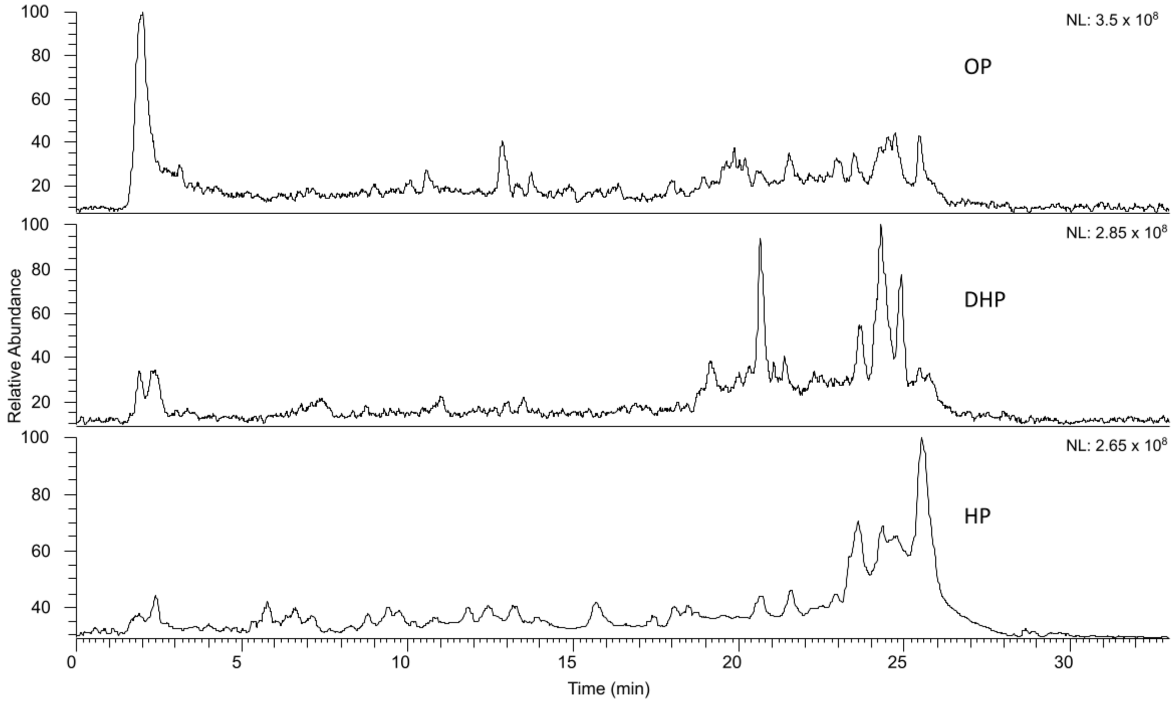

Figure S1: OP, DHP and HP samples total ion current (TIC) chromatograms in the full acquisition time range

Table S1: All Infected-discriminating features reported together with Rt (min)

Feature	RT med (min)	*m/z* [M-H]-med	Feature	RT med	*m/z* [M-H]-med
FT0220	7.27	219.0393	FT1947	2.06	569.1844
FT0249	23.20	226.0766	FT1956	20.50	570.1812
FT0305	15.21	242.0709	FT1972	14.09	572.2540
FT0489	7.21	297.0540	FT1980	16.86	573.2088
FT0534	11.16	305.0698	FT2018	11.11	581.2708
FT0577	20.54	314.0955	FT2034	22.66	583.2080
FT0664	23.21	331.1131	FT2044	9.10	585.2295
FT0672	11.63	333.1494	FT2057	2.11	589.2255
FT0740	18.93	348.1393	FT2067	10.31	591.2187
FT0759	20.58	351.1295	FT2075	21.34	593.3441
FT0761	23.22	351.1289	FT2077	9.87	594.1438
FT0806	1.97	360.1172	FT2079	21.32	594.3473
FT0814	1.95	362.1329	FT2084	20.13	595.1318
FT0819	11.53	363.1591	FT2092	11.76	596.2898
FT0828	17.98	364.1416	FT2190	10.70	621.2647
FT0833	19.42	365.1177	FT2194	10.63	622.2685
FT0845	16.05	367.1331	FT2214	14.33	627.2179
FT0847	22.69	367.1335	FT2228	20.49	632.1723
FT0852	13.02	368.1366	FT2312	24.61	668.2320
FT0870	3.67	373.0684	FT2326	19.31	671.6333
FT1011	24.70	396.1315	FT2330	12.26	673.2800
FT1062	20.11	406.1439	FT2416	24.67	707.1864
FT1081	3.10	408.1521	FT2433	21.81	713.1996
FT1089	9.65	409.2005	FT2461	17.11	724.2040
FT1093	19.34	411.1588	FT2478	11.43	727.3260
FT1097	10.54	411.2162	FT2490	20.08	731.2608
FT1111	2.06	414.1632	FT2540	13.03	751.2685
FT1131	20.18	418.1284	FT2564	9.69	755.2839
FT1133	13.44	419.1847	FT2574	21.86	757.2582
FT1159	19.68	425.1380	FT2582	21.38	759.2584
FT1228	21.10	439.1536	FT2596	21.50	765.2114
FT1234	21.09	440.1568	FT2621	12.19	775.2525
FT1254	24.80	444.1080	FT2622	12.48	775.3252
FT1347	13.30	460.2189	FT2646	24.77	783.2375
FT1366	10.02	463.1505	FT2654	24.82	785.2534
FT1373	22.27	464.1177	FT2688	12.24	801.2674
FT1386	16.33	465.2260	FT2695	10.64	803.2826
FT1398	18.61	467.2418	FT2718	19.92	811.2308
FT1399	8.15	467.2414	FT2784	23.79	843.4509
FT1403	6.87	469.1844	FT2791	21.87	848.2918
FT1407	24.79	470.1230	FT2817	10.66	863.3033
FT1435	9.48	475.1737	FT2827	22.88	871.4541
FT1457	16.27	478.1558	FT2833	13.16	876.3796
FT1474	14.71	481.2207	FT2853	24.24	888.2863
FT1494	19.13	484.1822	FT2863	13.03	893.3407
FT1512	9.85	487.2099	FT2872	16.71	896.1807
FT1552	8.77	495.1634	FT2876	19.31	897.1838
FT1601	11.20	506.1544	FT2912	10.66	921.3079
FT1614	22.57	509.1553	FT2917	19.84	923.2088
FT1634	15.15	512.2341	FT2920	19.84	924.2121
FT1672	20.90	519.6365	FT2947	24.19	945.2872
FT1675	19.81	520.1349	FT2968	21.58	971.3381

FT1691	7.67	523.1939	FT2979	20.89	980.2586
FT1705	15.98	525.2460	FT3012	14.10	1023.4675
FT1722	18.61	528.2652	FT3013	14.09	1024.4709
FT1792	17.09	540.2073	FT3065	21.92	1106.3245
FT1879	2.06	557.2002	FT3066	22.55	1107.3363
FT1895	16.69	561.2450	FT3068	23.19	1109.3428
FT1900	16.69	562.2485	FT3080	20.48	1141.3687

Table S2: All healthy-discriminating features reported together with Rt (min)

Feature	RT med (min)	m/z [M-H]$^-$ med	Feature	RT med	m/z [M-H]$^-$ med
FT0014	1.87	118.9295	FT0334	20.28	253.1402
FT0018	12.84	122.0312	FT0371	7.41	267.0438
FT0019	7.39	123.0435	FT0425	24.29	281.1476
FT0032	8.54	135.0433	FT0449	2.13	286.0732
FT0047	22.04	143.1058	FT0470	2.18	291.0286
FT0050	4.00	145.0849	FT0549	18.62	309.0898
FT0056	24.25	149.0589	FT0659	25.07	330.1871
FT0063	2.40	153.0172	FT0670	20.99	332.2463
FT0066	7.41	154.0567	FT0811	13.61	361.0862
FT0101	9.33	173.1156	FT0816	13.61	362.0895
FT0137	7.39	189.0293	FT0841	7.40	367.0353
FT0139	7.38	191.0263	FT0848	8.87	367.1329
FT0154	18.64	196.0664	FT0856	8.98	368.1210
FT0191	2.42	211.0575	FT0876	24.63	373.1236
FT0196	5.60	211.0576	FT0880	24.63	374.2565
FT0207	20.13	215.1253	FT1082	18.64	409.0453
FT0236	2.32	224.0821	FT1461	2.57	479.1335
FT0271	18.65	231.0389	FT1762	24.43	537.7061
FT0281	24.11	237.1221			
